# SGK1-FoxO1 Signaling Pathway Mediates Th17/Treg Imbalance and Target Organ Inflammation in Angiotensin II-Induced Hypertension

**DOI:** 10.3389/fphys.2018.01581

**Published:** 2018-11-15

**Authors:** Ya-Nan Du, Xiao-Feng Tang, Lian Xu, Wen-Dong Chen, Ping-Jin Gao, Wei-Qing Han

**Affiliations:** ^1^Shanghai Key Laboratory of Hypertension, Ruijin Hospital, Shanghai Jiao Tong University School of Medicine, Shanghai, China; ^2^Shanghai Institute of Hypertension, Shanghai, China; ^3^Laboratory of Vascular Biology, Institute of Health Sciences, Shanghai Institutes for Biological Sciences, Chinese Academy of Sciences, Shanghai, China

**Keywords:** serum/glucocorticoid regulated kinase 1, Th17 cells, Treg cells, target organ damage, angiotensin II

## Abstract

It has been demonstrated that serum/glucocorticoid regulated kinase 1 (SGK1) and the downstream transcription factor forkhead box O1 (FoxO1) plays a critical role in the differentiation of T helper 17 cells/regulatory T cells (Th17/Treg). In the present study, we hypothesized that this SGK1-FoxO1 signaling pathway is involved in Th17/Treg imbalance and target organ damage in angiotensin II (AngII)-induced hypertensive mice. Results show that SGK1 inhibitor EMD638683 significantly reversed renal dysfunction and cardiac dysfunction in echocardiography as indicated by decreased blood urine nitrogen and serum creatinine in AngII-infused mice. Flow cytometric assay shows that there was significant Th17/Treg imbalance in spleen and in renal/cardiac infiltrating lymphocytes as indicated by the increased Th17 cells (CD4^+^-IL17A^+^ cells) and decreased Treg cells (CD4^+^-Foxp3^+^). Consistently, real-time PCR shows that Th17-related cytokines including IL-17A, IL-23, and tumor necrosis factor α (TNF-α) was increased and Treg-related cytokine IL-10 was decreased in renal and cardiac infiltrating lymphocytes in AngII-infused mice. Meanwhile, SGK1 protein level, as well as its phosphorylation and activity, was significantly increased in spleen in AngII-infused rats. Furthermore, it was found that splenic phosphorylated FoxO1 was significantly increased, whereas total FoxO1 in nuclear preparation was significantly decreased in AngII-infused mice, suggesting that increased FoxO1 phosphorylation initiate its translocation from cytoplasm to nucleus. Notably, all changes were significantly inhibited by the treatment of EMD638683. These results suggest that SGK1 was involved in Th17/Treg imbalance and target organ damage in AngII-induced hypertension.

## Introduction

It has been demonstrated that T lymphocytes play an important role in inflammation and autoimmune diseases (Solak et al., 2016). CD4^+^ T lymphocytes, also called T helper cells (Th cells), include Th1, Th2, Th17, and regulatory T lymphocytes (Treg). Th1 cells defend against intracellular pathogens and certain bacteria by directing cell-mediated immunity. Th2 cells direct humoral antibody-mediated immunity fighting against extracellular pathogens and initiating allergic reactions (Yang et al., 2017). Th17 cells are highly pathogenic helper T cells, whereas Treg cells mainly mediate their immune-suppressive effects (Iwakura and Ishigame, 2006; Vignali et al., 2008). The differentiation of Th17 and Treg requires retinoid-related orphan receptor γt (RORγt) and forkhead box P3 (Foxp3), respectively, and their differentiation is mutually inhibitory. For example, cytokines that activate the transcription factor signal transducer and activator of transcription 3 (STAT3), such as interleukin-6 (IL-6), IL-21, and IL-23, promote Th17 differentiation and inhibit the generation of Treg cells, whereas STAT5-activating cytokines, such as IL-2, have the opposite effects (Noack and Miossec, 2014; Villarino and Laurence, 2015).

Accumulating data show that Th17/Treg imbalance, characterized by increased Th17 and decreased Treg, is involved in the initiation and development of hypertension including spontaneously hypertension, angiotensin II (AngII)-induced hypertension, and deoxycorticosterone acetate (DOCA)-salt hypertension. For example, it has been reported that Th17/Treg imbalance was present in spleen of spontaneously hypertensive rats (SHR), which was significantly inhibited by splenic sympathetic denervation (Katsuki et al., 2015). Madhur et al. (2010) reported that AngII infusion increased IL-17 production from T cells and IL-17 protein in the aortic media. AngII-induced blood pressure increase was significantly inhibited in IL-17 knockout mice. Furthermore, serum levels of Th17 in diabetic humans were significantly increased in hypertensive patients compared with normotensive subjects (Madhur et al., 2010). Another study shows that Th17 cells were increased and Treg cells were decreased in peripheral tissues, heart, and kidney of DOCA-salt-induced hypertensive rats, and that all these changes were significantly inhibited by aldosterone antagonist spironolactone and anti-IL-17 antibody (Amador et al., 2014). However, the mechanism underlying how Th17/Treg imbalance occurs in hypertension remains largely unknown.

The serum/glucocorticoid regulated kinase (SGK) family of serine/threonine kinases consists of three isoforms, SGK1, SGK2, and SGK3. This family of kinases is highly homologous to the protein kinase B or Akt (PKB/Akt) kinase family, sharing similar upstream activators and downstream targets. The catalytic domain of SGK is 54% identical with that of PKB, although lacking the PtdIns (3,4,5) P_3_-binding pleckstrin-homology domain, SGK retains the residues that are phosphorylated by 3-phosphoinositide dependent protein kinase 1/2 (PDK1/2), which are Thr^256^ and Ser^422^ in SGK (Kobayashi and Cohen, 1999). SGKs have been implicated in the regulation of cell growth, proliferation, survival, and migration (Bruhn et al., 2010). Recent studies show that SGK1 plays a critical role in the regulation of Th17/Treg differentiation in salt-induced inflammation and AngII-induced hypertension (Wu et al., 2013). For example, it has been reported that a modest increase in salt concentration induces SGK1 expression, promotes IL-23R expression, and enhances Th17 cell differentiation *in vitro* and *in vivo* (Wu et al., 2013), and that SGK1 signaling in T cells promotes hypertension and contributes to end-organ damage (Norlander et al., 2017).

Recent studies show that transcription factor forkhead box O1 (FoxO1) is phosphorylated by SGK1, and subsequently translocates from the nucleus to the cytosol during Th17/Treg differentiation (Fabre et al., 2005; Charvet et al., 2006). It has been shown that FoxO1 in the nucleus decreases Th17 generation *in vitro* as well as transcription of both IL-17A and IL-23R RORgt-target genes (Laine et al., 2015). However, FoxO1 in nucleus promotes Foxp3 expression by binding to Foxp3 conserved non-coding sequence 1 (CNS1) region (Wu et al., 2018). EMD638683 is a potent and highly selective SGK1 inhibitor. It has been shown EMD638683 alleviated cardiac inflammation partially by inhibiting nucleotide-binding oligomerization domain-like receptor with pyrin domain 3 (NLRP3) inflammasome activation (Gan et al., 2018). However, the mechanisms underlying SGK1-mediated inflammation remain largely unknown. In the present study, we hypothesized that SGK1-FoxO1 signaling pathway is involved in Th17/Treg imbalance, inflammation, and target organ damage in AngII-infused mice, and that inhibition of SGK1 with EMD638683 would attenuate these changes.

## Materials and Methods

### Antibodies and Reagents

Antibodies for PerCP/Cy5.5-conjugated CD4, Phycoerythrin (PE)-conjugated IL17A, and PE-conjugated Foxp3 were from BD Bioscience (San Jose, CA, United States), CD4 (L3T4) MicroBeads kits for magnetic-activated cell sorting (MACS) were from Miltenyi Biotec (Bergisch Gladbach, Germany), Ang II was from Sigma-Aldrich (St. Louis, MO, United States).

### Animals and Treatment

Male 8 to 10-week-old male C57/B6 mice were purchased from the Shanghai Experimental Animal Center, China. The investigation conforms with the Guide for the Care and Use of Laboratory Animals published by the United States National Institutes of Health (NIH Publication No. 85-23, revised 1996) and was approved by the Ethics Review Board of Ruijin Hospital, Shanghai, China. Four groups of animals were included: vehicle infusion (0.9% NaCl solution, control), vehicle infusion+EMD638683, AngII infusion, Ang II infusion+EMD638683. Ang II (1000 ng/kg/min) was infused for 2 weeks using Alzet mini-osmotic pumps (model 1002, DURECT, Cupertino, CA, United States) implanted subcutaneously in the surgery. EMD638383 was delivered i.p daily at a dosage of 10 mg/kg body weight. Systolic blood pressure was measured on every week by tail-cuff plethysmography (Visitech Systems, Apex, NC, United States) as a control for efficient AngII infusion. All surgical procedures were practiced under anesthesia.

### Echocardiography

After anesthetized with 3% isoflurane, mice were shaved and taped supine to electrocardiograph electrodes with ultrasound transmission gel on a heated procedure board as described previously (Li et al., 2014). Echocardiography was recorded with an 18–38 MHz linear-array transducer with a Vevo2100 Imaging System (Visual Sonics, Toronto, ON, Canada). To ensure consistence, all the recording and offline calculation were conducted by a single investigator who was blinded to animal groups. All M-mode measurements were performed in end-diastole and end-systole according to the leading edge-to-leading edge method of the American Society of Echocardiography. The parameters of the left ventricle (LV) included: intraventricular septum (IVS), left ventricular internal diameter (LVID), left ventricular posterior wall (LVPW), ejection fraction (EF), and fractional shortening (FS) (Li et al., 2014).

### Histology

Cardiac and kidney tissue samples were fixed in 4% paraformaldehyde, embedded in paraffin, and sectioned at 5 μm intervals. Masson trichrome staining was performed using standard procedures. Sections were stained with Masson trichrome for the evaluation of fibrosis. Images were captured using a Nikon Labophot 2 microscope equipped with a Sony CCD Iris/RGB color video camera attached to a computerized imaging system, and analyzed with ImagePro Plus 3.0.

### RNA Analysis

Total RNA was extracted by the Trizol reagent method, and cDNA was generated using the Takara Qpcr RT kit (Takara Bio, Shiga, Japan). All reactions were performed with SYBR Premix Ex TaqII (Takara Bio, Shiga, Japan) and Applied Biosystems 7900 Real-time PCR system (Applied Biosystems-Life Technologies, Grand Island, NY, United States) according to the manufacturer’s protocol. RT-PCR primers are shown in Table 1.

**Table 1:** Primer used for real-time PCR.

Gene	Accession no.	Forward	Reverse
Foxp3	AY357712	CCCATCCCCAGGAGTCTTG	ACCATGACTAGGGGCACTGTA
GAPDH	NM_008085	TGGATTTGGACGCATTGGTC	TTTGCACTGGTACGTGTTGAT
IL-10	NM_010548	GCTCTTACTGACTGGCATGAG	CGCAGCTCTAGGAGCATGTG
IL-17A	NM_010552	TTTAACTCCCTTGGCGCAAAA	CTTTCCCTCCGCATTGACAC
IL-23	NM_031252	ATGCTGGATTGCAGAGCAGTA	ACGGGGCACATTATTTTTAGTCT
RORγt	NM_011281	GACCCACACCTCACAAATTGA	AGTAGGCCACATTACACTGCT
SGK1	BC070401	TGGAAAGGTTCTTCTGGCTAGG	CACCAGGAAAGGGTGCTTCA
SGK2	NM_134463.1	AGGCACTGACACTTGGCTCTTG	AGGCACTGACACTTGGCTCTTG
SGK3	XM_017593903.1	TGCTGAGATTGCCAGTGCCTTG	TGCTGAGATTGCCAGTGCCTTG
TNF-α	NM_013693	CCCTCACACTCAGATCATCTTCT	GCTACGACGTGGGCTACAG

### Western Blot

Total and nuclear protein preparation from spleen and western blotting were performed as described previously (Han et al., 2013). In brief, after boiling for 5 min at 95°C in loading buffer, protein were subjected to SDS–PAGE. For total protein, the membrane was probed with primary anti-SGK1 antibody (AbSci, Vancouver, WA, Canada), anti-phosphorylated SGK1 (Ser422) antibody (AbSci, Vancouver, WA, Canada), anti-phosphorylated FoxO1 antibody (ab52857, Abcam, Cambridge, MA, United States), and developed with horseradish peroxidase (HRP)-labeled secondary antibody. GAPDH was detected by using HRP-labeled anti-GAPDH antibody (1:5000, Santa Cruz Biotechnology, Dallas, TX, TX), and used as a loading control for total protein. For nuclear protein, the membrane was probed with anti-FoxO1 antibody (ab131339, Abcam, Cambridge, MA, United States), and developed with HRP-labeled secondary antibody (1:2000). Transcription factor II D (TFIID) was detected using primary HRP-labeled anti-TFIID antibody (SC-421 HRP, Santa Cruz Biotechnology, Dallas, TX, United States), and used as a loading control for nuclear protein (Han et al., 2013). The immunoreactive bands were detected by chemiluminescence and developed on Kodak Omat X-ray films. X-ray films were analyzed using the ImageJ software (NIH, Bethesda, MD, United States).

### SGK1 Kinase Assays

Then SGK1 activity in lymphocytes was measure using SGK Kinase Assay Kits (Immunechem Pharmaceuticals Inc., Burnaby, BC, Canada) as described in the user instruction. Briefly, anti-SGK1 antibody was incubated with protein A agarose beads and cell lysate. Then bovine serum albumin (BSA)-kinase substrate and adenosine triphosphate (ATP) was added to start the kinase reaction at 33°C for 1 h, the kinase reaction was terminated by boiling in SDS-sample loading buffer. Western blot was performed as described above with primary anti-phosphopeptide substrate (RPRAApTF-NH2) antibodies. Equal amount of protein was probed with GAPDH as loading control.

### Isolation of Splenocytes and Infiltrating Lymphocytes From Kidney and Heart

The spleen was removed and triturated in phosphate buffer saline (PBS) with 5% fetal bovine serum (FBS) (Gibco-Life Technologies) at 4°C. The cell suspension was pipetted through a nylon cell strainer (40 μm, BD Falcon, San Jose, CA, United States) into a fresh tube and centrifuged at 300 *g* for 5 min at 4°C. Erythrocytes were also lysed with red cell lysing buffer for 10 min at room temperature and centrifuged at 300 *g* for 5 min at 4°C (Katsuki et al., 2015).

Kidney and heart tissues were cut into small pieces and digested in collagenase I (kidney, 1 mg/ml) or collagenase II (heart, 1 mg/ml) dissolved in RPMI1640 for 30 min at 37°C. The tissue debris was pipetted through a nylon cell strainer (40 μm, BD Falcon, San Jose, CA, United States) into a fresh tube and centrifuged at 300 *g* for 5 min at 4°C, and the digested cells were isolated using Percoll-Paque density centrifugation after homogenization (Yang et al., 2012).

### Flow Cytometric Analysis

Isolated cell suspensions were incubated and stained for fluorescent-activated cell sorting (FACS) analysis, Fluorescence data were collected using an EPICS XL Flow Cytometer (Beckman Coulter, Brea, CA, United States) with PerCP-Cy5.5-coupled CD4^+^ antibody and PE-coupled IL17/Foxp3, respectively, data analysis was performed with the aid of computer software (FlowJo 10.0). Th17 cells were analyzed after stimulation of the cells with PMA and ionomycin in the presence of GolgiStop. Lymphocytes were gated based on their low forward scatter and side scatter property, Th17 cells was presented as CD4^+^-IL17A^+^ cells and Treg cells was presented as CD4^+^-Foxp3^+^ cells.

### Measurement of Serum Pro-inflammatory Cytokines, Anti-inflammatory Cytokines, Serum Creatinine, Blood Urine Nitrogen

Serum of all groups were collected and stored in -80°C before analysis. Serum creatinine and blood urea nitrogen (BUN) were measured with fully automatic biochemical instruments. Enzyme-linked immunosorbent assay (ELISA) was performed according to the manufactures’ instructions of the ELISA kits (R&D Systems). The OD value at 450 nm was measured. The concentrations of IL-17, IL-23, TNF-α, and IL-10 were calculated according to the standard curve (Chen et al., 2013).

### Statistical Analysis

Results are expressed as mean ± standard error of the mean (SEM). Differences were analyzed by one way ANOVA (Figures 1–8), and a *P*-value less than 0.05 was considered statistically significant.

**FIGURE 1:**
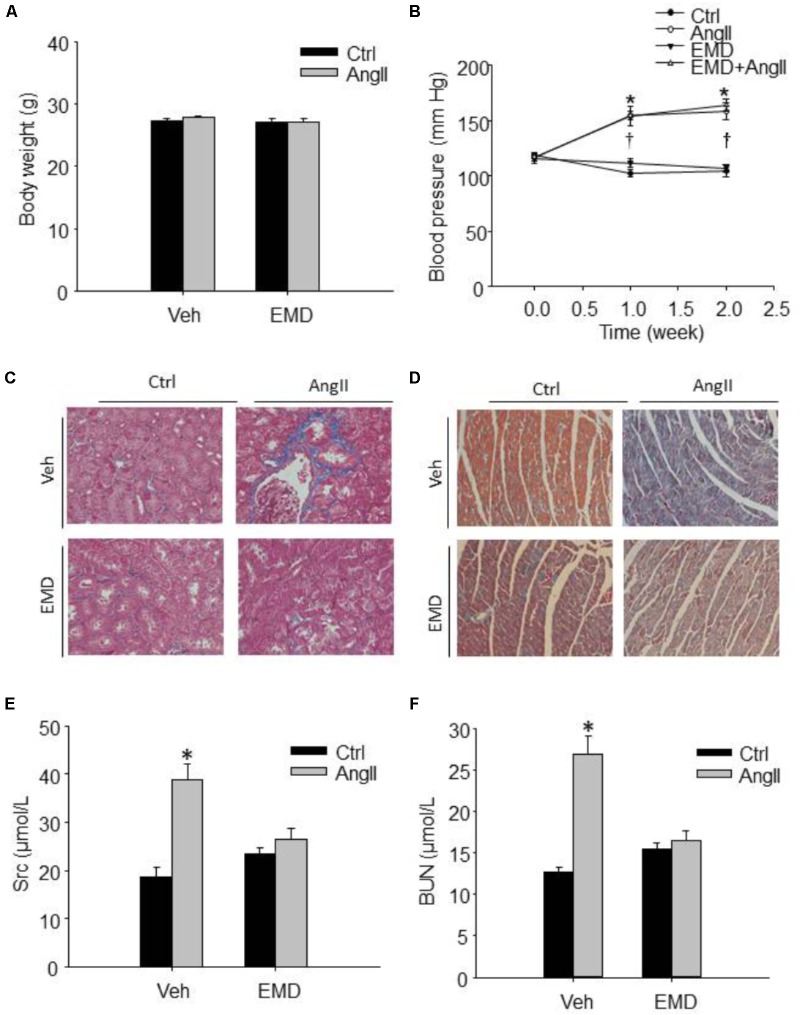
Effect of SGK1 inhibitor EMD638683 on blood pressure, body weight, renal, and cardiac fibrosis in AngII-infused mice. Summarized data showing the changes of blood pressure **(A)** and body weight **(B)** in sham control and AngII-infused mice with or without EMD638683 treatment. Representative Masson staining showing the effect of EMD638683 on renal **(C)** and cardiac fibrosis **(D)** in AngII-induced mice. To evaluate the effect of EMD638683 on renal function, the changes of serum creatinine **(E)** and BUN **(F)** was measured in sham control and AngII-infused mice with or without EMD638683 treatment. EMD, EMD638683. *n* = 8–10 mice. ^∗^*P* < 0.05 vs. other groups in **(E,F)**, ^∗^*P* < 0.05 vs. control group and ^†^*P* < 0.05 vs. EMD638683 group in **(B)**.

**FIGURE 2:**
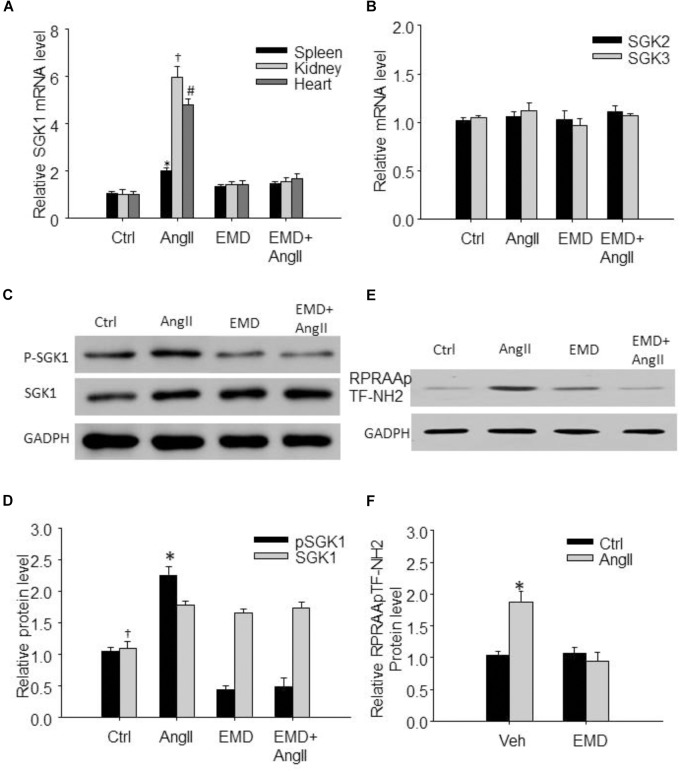
Increased expression of SGK1 in spleen and in renal and cardiac infiltrating lymphocytes in AngII-infused mice. **(A)** Summarized data showing relative SGK1 mRNA level in spleen and in infiltrating lymphocytes isolated from kidney and heart from sham control and AngII-infused mice with or without EMD638683 treatment. **(B)** Summarized data showing relative SGK2 and SGK3 mRNA level in spleen from sham control and AngII-infused mice with or without EMD638683 treatment. Representative western blot **(C)** and summarized data **(D)** showing pSGK1 and SGK1 protein level in spleen from sham control and AngII-infused mice with or without EMD638683 treatment. RPRAATF-NH2 was used as a substance in SGK1 kinase activity measurement, the phosphorylated product RPRAApTF-NH2 was measured with western blot to indicate SGK1 kinase activity. Representative western blot **(E)** and summarized data **(F)** showing RPRAApTF-NH2 level in splenic lysate from sham control and AngII-infused mice with or without EMD638683 treatment. GAPDH was used as loading control. EMD, EMD638683. *n* = 8–10 mice. ^∗^*P* < 0.05, ^†^*P* < 0.05, and ^#^*P* < 0.05 vs. other groups for spleen, kidney, and heart, respectively, in **(A)**. ^∗^*P* < 0.05, ^†^*P* < 0.05, and ^#^*P* < 0.05 vs. other groups for pSGK1 and SGK1, respectively, in **(B)**.

**FIGURE 3:**
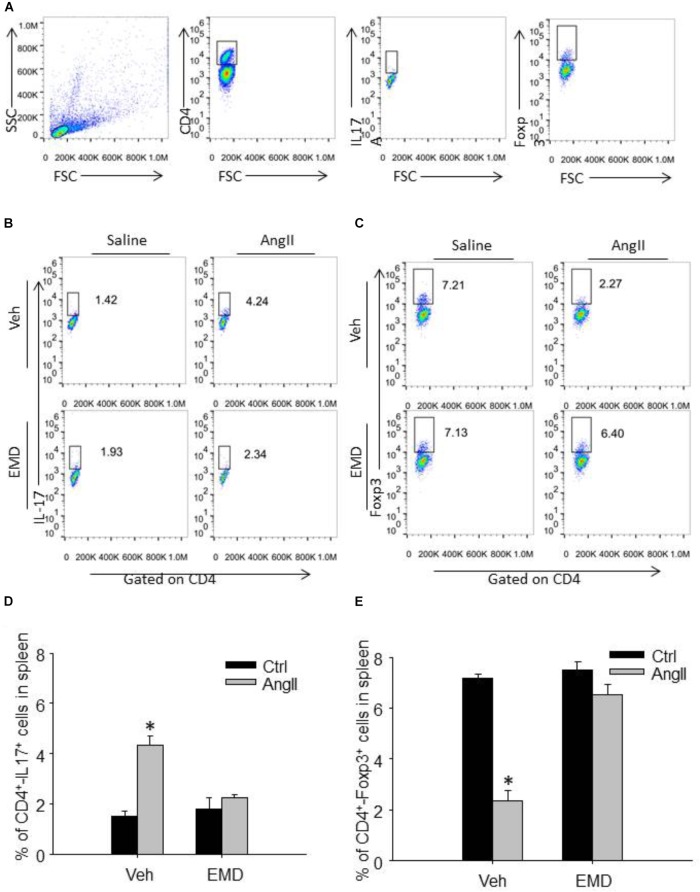
Effect of EMD638683 on splenic Th17 and Treg cells in AngII-infused mice. **(A)** Schema for flow cytometric identification of splenic, renal and cardiac CD4^+^-Th17^+^ T cells (Th17 cells) and CD4^+^-Foxp3^+^ T cells (Treg cells), expressed as a percentage of the lymphocyte gate of the spleen determined from side and forward scatter (SSC, FSC) plots. Representative FACS profiles of Th17 cells **(B)** and Treg cells **(C)** as a percentage of CD4^+^ cells in the spleen. Summarized data showing the percentage of Th17 cells **(D)** and Treg cells **(E)** as a percentage of CD4^+^ cells in the spleen. EMD, EMD638683. *n* = 8–10 mice. ^∗^*P* < 0.05 vs. other groups.

**FIGURE 4:**
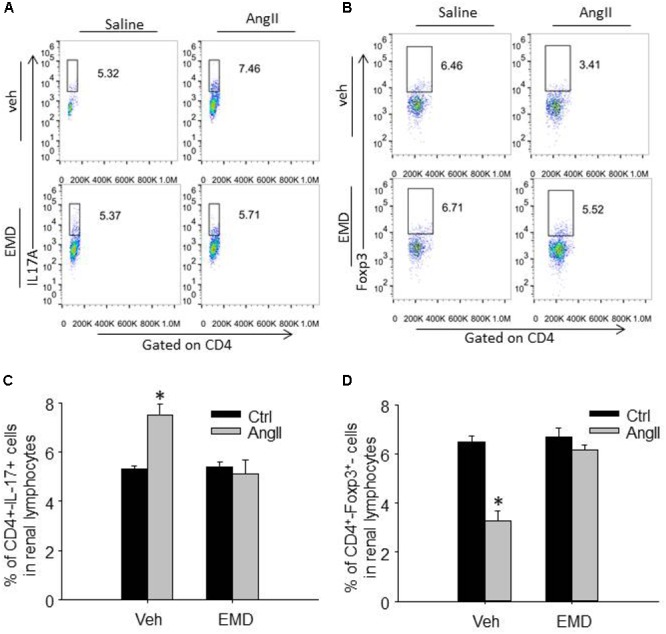
Effect of EMD638683 on renal Th17 and Treg cells in AngII-infused mice. Representative FACS profiles of Th17 cells **(A)** and Treg cells **(B)** as a percentage of CD4^+^ cells in the kidney. Summarized data showing the percentage of Th17 cells **(C)** and Treg cells **(D)** as a percentage of CD4^+^ cells in the kidney. EMD, EMD638683. *n* = 8–10 mice. ^∗^*P* < 0.05 vs. other groups.

**FIGURE 5:**
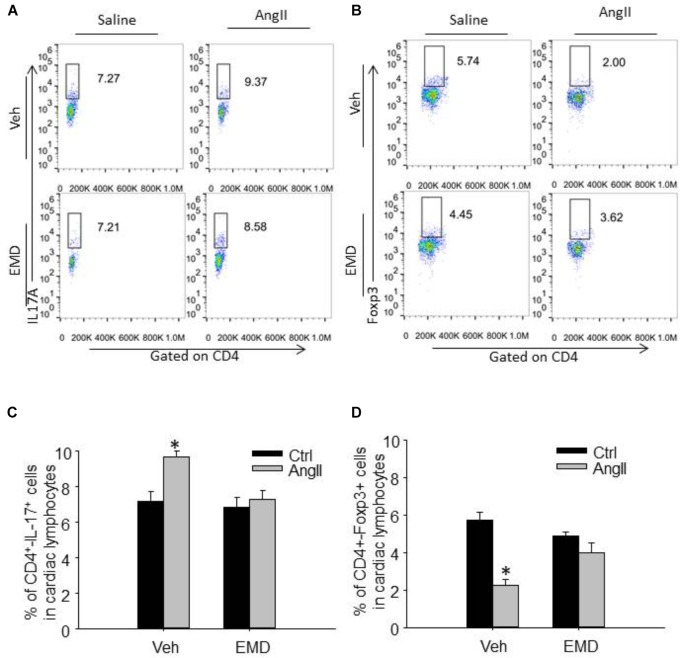
Effect of EMD638683 on cardiac Th17 and Treg cells in AngII-infused mice. Representative FACS profiles of Th17 cells **(A)** and Treg cells **(B)** as a percentage of CD4^+^ cells in the heart. Summarized data showing the percentage of Th17 cells **(C)** and Treg cells **(D)** as a percentage of CD4^+^ cells in the heart. EMD, EMD638683. *n* = 8–10 mice. ^∗^*P* < 0.05 vs. other groups.

**FIGURE 6:**
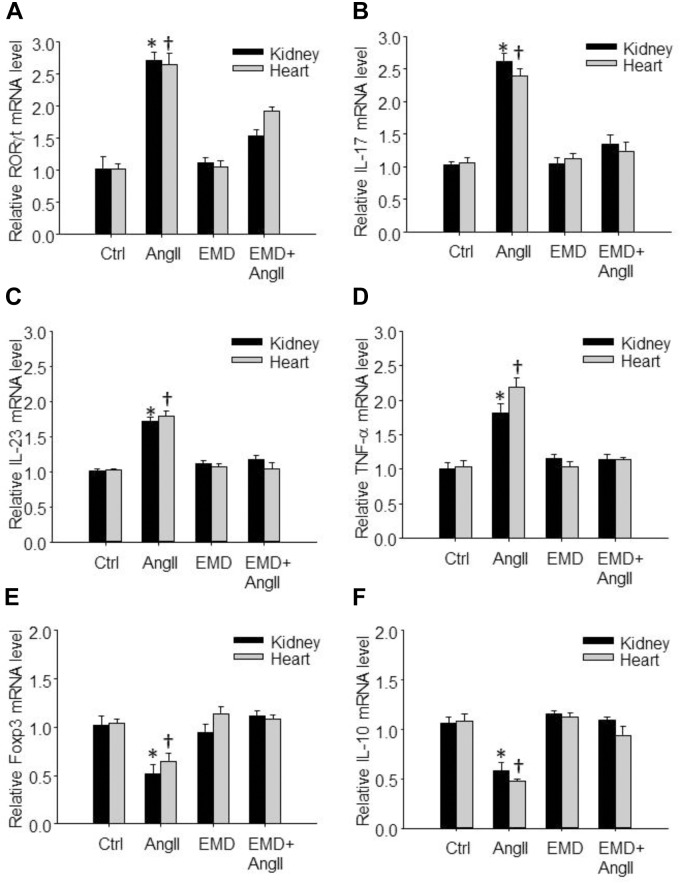
Effect of EMD638683 on renal and cardiac inflammation in AngII-infused mice. Summarized data showing mRNA expression of Th17- and Treg-related transcription factors and cytokines including RORγt **(A)**, IL-17 **(B)**, IL-23 **(C)**, TNF-α **(D)**, Foxp3 **(E)**, and IL-10 **(F)** in kidney and heart from sham control and AngII-infused mice with or without EMD638683 treatment. EMD, EMD638683. *n* = 8–10 mice. ^∗^*P* < 0.05 and ^†^*P* < 0.05 vs. other groups for kidney and heart, respectively.

**FIGURE 7:**
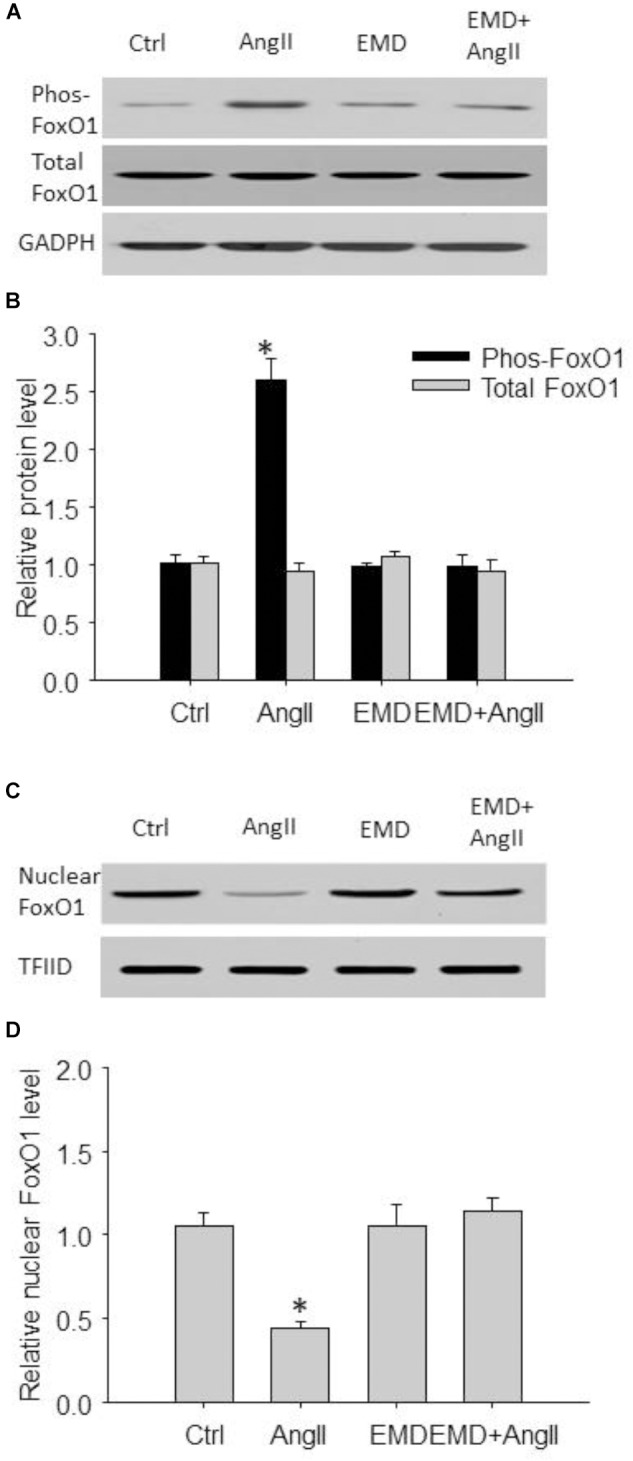
Inhibitory effect of EMD638683 on AngII-induced FoxO1 phosphorylation and its translocation from nucleus to cytoplasm in mouse splenic cells. Representative western blot **(A)** and summarized data **(B)** showing splenic phosphorylated FoxO1 level and total FoxO1 level in sham control and AngII-infused mice with or without EMD638683 treatment. Representative western blot **(C)** and summarized data **(D)** showing splenic nuclear FoxO1 level in sham control and AngII-infused mice with or without EMD638683 treatment. EMD, EMD638683. *n* = 4–5 mice. ^∗^*P* < 0.05 vs. other groups for pFoxO1.

**FIGURE 8:**
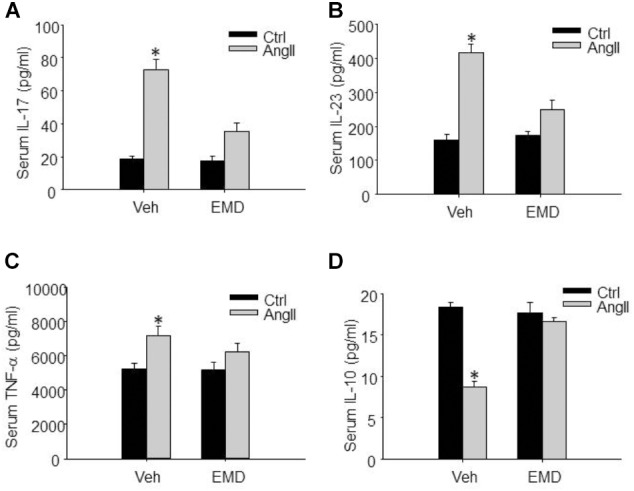
Inhibitory effect of EMD638683 on serum proinflammatory and anti-inflammatory cytokines in AngII-infused mice. Summarized data showing the changes of serum proinflammatory cytokines IL-17 **(A)**, IL-23 **(B)**, TNF-α **(C)**, and anti-inflammatory cytokine IL-10 **(D)** in sham control and AngII-infused mice with or without EMD638683 treatment. EMD, EMD638683. *n* = 8–10 mice. ^∗^*P* < 0.05 vs. other groups.

## Results

### Effect of EMD638683 on Blood Pressure, Renal, and Cardiac Fibrosis in AngII-Infused Mice

Figure 1A shows that there is no significant difference in body weight in the four groups of mice. In contrast, blood pressure was significantly increased in AngII-infused mice, and that EMD683638 did not show significant effect on blood pressure (Figure 1B). These results suggest that SGK1 was not involved in blood pressure increase in AngII-infused mice.

Masson trichrome staining was performed to evaluate the effect of EMD638683 on collagen deposition in kidney and heart, it was found that AngII infusion significantly increased collagen deposition in kidney and heart, which was significantly inhibited by EMD-638683 (Figures 1C,D) (Relative renal collagen: Con, 1.08 ± 0.17; AngII, 2.45 ± 0.32; EMD, 1.22 ± 0.27; EMD+AngII, 1.23 ± 0.21. Relative cardiac collagen: Con, 1.06 ± 0.12; AngII, 3.21 ± 0.51; EMD, 1.35 ± 0.18; EMD+AngII, 1.19 ± 0.07. *P* < 0.05 AngII group vs. other group in both renal and cardiac collagen deposition). These results suggest that SGK1 activation may contribute to target organ damage in AngII-infused hypertension.

### Effect of EMD638683 on Renal Function and Cardiac Hypertrophy in AngII-Infused Mice

Next, serum creatinine and BUN levels were measured to evaluate the changes of renal function. As shown in Figures 1E,F, serum creatinine and BUN were significantly increased in AngII-infused mice, which were significantly inhibited in AngII-infused mice treated with EMD638683. These results suggest that SGK1 plays a role in renal dysfunction in AngII-induced hypertension.

The protective effect of EMD638683 on heart function was evaluated by ultrasound measurement. As shown in Table 2, LVPW and LV mass significantly increased in AngII-infused mice compared with control. In contrast, LVID and LV volume significantly decreased in AngII-infused mice compared with control. Notably, these changes were effectively inhibited in AngII-infused mice treated with EMD638683, suggesting that SGK1 was involved in AngII-induced cardiac hypertrophy in mice.

**Table 2:** Effect of EMD638683 on heart function in AngII-infused mice by using echocardiography.

	Control	AngII	EMD638683	AngII+EMD638683
IVS; d, mm	0.93 ± 0.03	1.18 ± 0.08*	0.92 ± 0.04	1.01 ± 0.05
IVS; s, mm	1.64 ± 0.051	1.51 ± 0.12	1.26 ± 0.08	1.33 ± 0.10
LVID; d, mm	3.62 ± 0.06	2.98 ± 0.27*	3.85 ± 0.22	3.59 ± 0.20^†^
LVID; s, mm	2.31 ± 0.31	1.86 ± 0.32*	2.83 ± 0.35	2.45 ± 0.23^†^
LVPW; d, mm	0.81 ± 0.02	1.18 ± 0.23*	0.78 ± 0.05	0.93 ± 0.08^†^
LVPW; s, mm	1.19 ± 0.13	1.43 ± 0.15*	1.00 ± 0.10	1.11 ± 0.09^†^
% EF	68.8 ± 7.03	68.9 ± 5.82	52.8 ± 6.41	60.71 ± 6.68
% FS	41.1 ± 7.31	39.0 ± 4.89	37.8 ± 4.37	37.08 ± 4.30
LV Mass, mg	92.6 ± 3.56	107.6 ± 17.1*	96.2 ± 4.02	86.80 ± 4.38^†^
LV Vol; d, μl	56.8 ± 1.65	36.4 ± 7.32*	65.4 ± 6.96	59.19 ± 5.76^†^
LV Vol; s, μl	33.2 ± 3.87	23.1 ± 4.38*	33.5 ± 6.97	33.62 ± 4.70^†^

*IVS, intraventricular septum; LVID, left ventricular internal diameter; LVPW, left ventricular posterior wall; EF, ejection fraction; FS, fractional shortening; LV, left ventricular; Vol, volume. *n* = 6–8 mice. ^∗^*P* < 0.05 vs. control, ^†^*P* < 0.05 vs. AngII group*.

### Effect of EMD638683 on the Expression and Phosphorylation of SGK1 in Spleen in AngII-Induced Mice

Previous studies show that SGK1 was increased in response to AngII stimulation, we then evaluate whether SGK1 was increased in spleen and in renal and cardiac infiltrating lymphocytes in AngII-infused mice. As shown in Figure 2A, real-time PCR results show that SGK1 mRNA level was significantly increased in splenic, renal, and cardiac infiltrating lymphocytes from AngII-infused mice compared with these from control rats, which was significantly inhibited by EMD638683. As shown in Figures 2C,D, western blot shows that SGK1 protein level and its phosphorylation were significantly increased in spleen. Furthermore, as indicated by RPRAAp TF-NH2 levels, SGK1 activity was significantly increased in spleen from AngII-infused mice from that from control mice (Figures 2E,F). Notably, the increased SGK1 phosphorylation and activity in spleen were significantly inhibited in AngII-infused mice treated with EMD638683. In contrast, there were no significant different in SGK2 and SGK3 mRNA in spleen among the four groups of mice (Figure 2B), suggesting that neither of them may not be involved in AngII-induced hypertension.

### EMD638683 Prevented Th17/Treg Imbalance in Spleen and Infiltrating Lymphocytes in AngII-Induced Mice by Flow Cytometry

We then further evaluated whether inhibition of SGK1 would prevent Th17/Treg imbalance in AngII-infused mice by using flow cytometry. Th17 cells were defined as CD4^+^-IL17^+^ cells, and Treg cells were defined as CD4^+^-Foxp3^+^ cells (Figures 3A,B). The collected spleen cells were directly used for flow cytometry since there are plenty of CD4^+^ cells. As shown in Figures 3C–F, the staining of Th17 was increased significantly whereas Treg staining was decreased significantly in spleen cells in AngII-infused mice compared with control mice, leading to Th17/Treg imbalance. In spleen cells from AngII-infused mice treated with EMD638683, however, the Th17/Treg imbalance was significant inhibited. These results suggest that SGK1 was involved in Th17/Treg imbalance in spleen.

To evaluate infiltrating Th17 and Treg in target organ, lymphocytes were collected from kidney and heart and used for flow cytometry. The representative flow cytometric images in Figures 4A,B and summarized data in Figures 4C,D show that the ratio of Th17 cells were significantly increased in kidney from AngII-infused mice compared with that from control mice. Similarly, the ratio of Th17 cells were significantly increased in heart from AngII-infused mice compared with that from control mice (Figures 5A–D). As expected, the Th17/Treg imbalance was lost in lymphocyte isolated from kidney and heart in AngII-infused mice treated with EMD638683. These results suggest that SGK1 was involved in Th17/Treg imbalance in spleen, as well as infiltrating Th17/Treg in kidney and heart in AngII-infused mice.

### EMD638683 Prevented the Expression of Th17- and Treg-Related Transcription Factors and Cytokines in Infiltrating Lymphocyte in AngII-Infused Mice

We then further evaluated whether EMD638683 would have any effect on Th17- and Treg-related transcription factors and cytokines by real-time PCR. Results show that the expression of Th17-related transcription factor RORγt and cytokines including IL-17, IL-23, and TNF-α was significantly increased in lymphocytes isolated from kidney and heart in AngII-infused mice (Figures 6A–D). In contrast, the expression of Treg-related transcription factor Foxp3 and cytokine IL-10 was significantly decreased in lymphocytes isolated from kidney and heart in AngII-infused mice (Figures 6E,F). Notably, the changed expression of these genes was significantly inhibited by EMD638683. These results suggest that SGK1 was involved in Th17/Treg-related inflammatory effects in target organ.

### EMD638683 Inhibited FoxO1 Phosphorylation and Its Translocation From Nuclear to Cytoplasm in Spleen

We then evaluated FoxO1 phosphorylation and its subcellular location by western blot. As shown in Figures 7A,B, the phosphorylated FoxO1 was significantly increased in spleen from AngII-induced hypertensive mice compared with that from control mice. In contrast, Figures 7C,D shows that total FoxO1 in the nuclear preparation was significantly decreased in spleen from AngII-induced hypertensive mice compared with that from control mice. Notably, these changes were significantly attenuated in AngII-infused mice treated with EMD638683, suggesting that FoxO1 phosphorylation and its translocation from nucleus to cytoplasm may be involved in Th17/Treg imbalance in splenic lymphocytes in AngII-induced hypertensive mice.

### EMD638683 Decreased Inflammatory Factors in the Peripheral Immune System

Finally, we evaluated whether SGK1 inhibition would improve peripheral immune system and prevent renal dysfunction, since previous studies show that peripheral immune system is changed and contributes to blood pressure increase and target organ damage in AngII-induced hypertension. It was found that serum IL-17, IL-23, and TNF-α was significantly increased, whereas serum anti-inflammatory factor IL-10 was significantly decreased in AnII-infused mice (Figures 8A–D). As expected, these changes were significantly inhibited by EMD638683 treatment. These results suggest that SGK1-mediated Th17/Treg imbalance may contribute to inflammation in peripheral immune system in AngII-induced hypertension.

## Discussion

In the present study, we evaluated whether SGK1-FoxO1 signaling pathway is involved in Th17/Treg imbalance in AngII-induced hypertension. It was found that SGK1 inhibitor EMD638683 significantly prevented renal function and cardiac hypertrophy, as well as renal and cardiac fibrosis, in AngII-infused mice. Notably, flow cytometry assay shows that there was obvious Th17/Treg imbalance in splenic lymphocytes and renal/cardiac infiltrating lymphocytes. Meanwhile, splenic SGK1 expression, as well as its phosphorylation and activity, was significantly increased. Furthermore, it was found that splenic phosphorylated FoxO1 was significantly increased, whereas total FoxO1 in nuclear preparation was significantly decreased in AngII-infused mice. Treatment with EMD638683 significantly inhibited these changes in AngII-infused mice, suggesting that SGK1-FoxO1 signaling pathway is involved in Th17/Treg imbalance and target organ damage in AngII-infused mice.

Firstly, the present study shows that inhibition of SGK1 with EMD638683 did not show significant effect on blood pressure increase in AngII-infused mice, which is consistent with a previous study showing that 4 weeks treatment with EMD638683 did not decrease high blood pressure in AngII-induced mice (Gan et al., 2017). However, this finding is in contrast to the finding that 4 weeks EMD638683 treatment decreased blood pressure in high salt-induced hypertension (Ackermann et al., 2011). These results suggest that SGK1 may play a different role in the regulation of blood pressure in different hypertensive animal models.

It has been well documented that SGK1 was involved renal function and cardiac fibrosis in DOCA salt- and AngII-induced hypertension. For example, it has been reported that SGK1 was involved in salt reabsorption, inflammation and blood pressure in salt-sensitive hypertension (Aoi et al., 2007; Xi et al., 2014). Recent studies suggest that SGK1 mediates AngII-induced sodium reabsorption via sodium-hydrogen exchanger-3 (NHE3) expression and Na^+^ reabsorption in proximal tubular cells (Vallon et al., 2006; Stevens et al., 2008), and renal fibrosis by stimulating connective tissue growth factor (CTGF) expression in human kidney fibroblasts (Hussain et al., 2008). Furthermore, it has been shown that SGK1 contributes to cardiac inflammation and fibrosis by mediating nucleotide-binding oligomerization domain-like receptor with pyrin domain 3 (NLRP3) inflammasome activation (Gan et al., 2018), and cardiac fibroblast-to-myofibroblast transition in AngII-induced hypertension (Yang et al., 2012). In addition, it has been reported that SGK1 plays a critical role in heart failure in transverse aortic constriction animal model (Das et al., 2012). In consistent with these findings, the present study shows that SGK1 was involved in AngII-induced renal and cardiac fibrosis.

Accumulating data show that pharmacological treatment targeting Th17/Treg imbalance provides beneficial effect on inflammation and fibrosis in various diseases. For example, Yodoi et al. (2015) reported that injection of recombinant mouse IL-2/anti-IL-2 monoclonal antibody complex selectively expanded Foxp3^+^ Treg, and decreased the incidence (52%) and mortality (17%) in AngII-induced aortic aneurysm formation in mice. Immunohistochemical analysis showed reduced accumulation of macrophages and increased numbers of Foxp3^+^ Treg in aneurysmal tissues, suggesting that expansion of Tregs may suppress local inflammation in the vessel wall and provide protection against abdominal aortic aneurysm formation (Yodoi et al., 2015). Meng et al. (2014) reported that Treg treatment markedly decreased macrophage and CD4^+^ T-cell infiltration and preserved the medial smooth muscle cells. Furthermore, Tregs decreased the levels of proinflammatory cytokines, matrix metalloproteinase-2 (MMP-2) and MMP-9, increased the expression of anti-inflammatory IL-10 and transforming growth factor β1 (TGF-β1), and suppressed apoptosis and oxidative stress (Meng et al., 2014). Th17 cells are highly pathogenic helper T cells that produce IL-17, IL-17F, IL-6, and TNF-α (Iwakura and Ishigame, 2006), whereas Treg cells mainly mediate their immune-suppressive effect via the suppression of inhibitory cytokine expression, such as IL-10 and TGF-β1 (Vignali et al., 2008).

It has been shown that high salt induced SGK1 expression, which lead to increased Th17 differentiation and decreased Treg differentiation, contributing to high salt-induced Th17/Treg imbalance (Wu et al., 2013; Binger et al., 2015). Importantly, it has been reported that the infiltration of the kidney by T lymphocytes was a prominent feature of AngII-dependent renal injury, and these damage effects including T cell infiltration and kidney damage were significantly inhibited by immune inhibitor mycophenolate mofetil (Crowley et al., 2008; Rodriguez-Iturbe, 2016). In the present study, we demonstrated that Th17/Treg imbalance is present in spleen and infiltrating T lymphocytes in kidney and heart, concomitant with the changes of Th17/Treg-related cytokines including IL-17, IL-23, and TNF-α in kidney and heart. It has been reported that SGK1 signaling in T cells promotes hypertension and contributes to end-organ damage (Norlander et al., 2017). In consistent with these findings, the present study shows that the expression and activity of SGK1 were increased in spleen and infiltrating lymphocytes in kidney and heart. The decreased SGK1 phosphorylation by EMD638683 may be responsible for AngII-induced SGK1 activation as reported previously (Gan et al., 2018). Surprisingly, EMD638683 decreased SGK1 mRNA level but not SGK1 protein level. This finding is consistent with a previous study showing that NADPH oxidase apocynin decreased mRNA level of gp91*^phox^*, a NADPH oxidase subunit, although gp91*^phox^* was not evaluated (Kanegae et al., 2010). Therefore, it seems that this phenomenon is not unique for EMD638683. The underlying mechanism, however, remains unknown. The finding that SGK2 and SGK3 were not changed in AngII-infused mice is consistent with previous studies demonstrating that both of them remain unchanged in AngII-infused mice (Gan et al., 2018).

Furthermore, the present study shows that FoxO1 phosphorylation and its translocation from nucleus to cytoplasm were significantly increased in spleen from AngII-infused mice. These findings are consistent with previous studies showing that FoxO1 phosphorylation by SGK1 and its translocation from the nucleus to the cytosol plays a critical role in the regulation of Th17/Treg balance (Fabre et al., 2005; Charvet et al., 2006; Wu et al., 2018). Notably, EMD638683 blocked the increased SGK1 phosphorylation, SGK1 activation, Th17/Treg imbalance, and the alteration of Th17/Treg-related cytokines. Therefore, these results suggest that SGK1-FoxO1 signaling pathway plays a critical role in Th17/Treg imbalance in AngII-induced hypertension.

Furthermore, the present study demonstrated that SGK1 inhibitor EMD638683 significantly inhibited the increased peripheral cytokines in AngII-induced hypertensive mice. This finding is consistent with previous studies showing that peripheral cytokines such as IL-17, IL-6, IL-1β, Interferonγ (IFN-γ), monocyte chemoattractant protein 1 (MCP-1), vascular endothelial growth factor (VEGF), and TNF-α were increased and contributed to target organ damages in AngII-induced hypertension in mice (Lee et al., 2006; Sanchez-Lemus et al., 2009; Madhur et al., 2010; Mirhafez et al., 2014; Park et al., 2017). For example, it has been shown that IL-17 production from T cells and IL-17 protein in the aortic media, and that the IL-17 deficiency displayed preserved vascular function, decreased superoxide production, and reduced T-cell infiltration in response to angiotensin II (Madhur et al., 2010). Lee et al. (2006) reported that blood pressure increases was significantly inhibited in IL-6 knockout mice. Mirhafez et al. (2014) reported that inflammatory factors including IFN-γ, TNF-α, MCP-1 was increased whereas IL-10 was decreased in serum from hypertensive patients compared with normotensive control. These studies were consistent with the present study showing that peripheral cytokines was increased in AngII-induced hypertension which may contribute to target organ damage.

In summary, the present study demonstrated that SGK1-FoxO1 signaling pathway is involved in Th17/Treg imbalance and contributes to renal/cardiac inflammation and fibrosis in AngII-induced hypertensive mice. Considering the important role of Th17/Treg imbalance, this finding may provide pharmacological target for the prevention and treatment of target organ damage associated with hypertension.

## Author Contributions

W-QH and P-JG designed the work. Y-ND, X-FT, LX, W-DC, and W-QH performed the study. Y-ND, X-FT, LX, W-DC, W-QH, and P-JG analyzed the data. Y-ND, X-FT, and LX drafted the manuscript. W-QH critically revised the manuscript, contributed to the final approved version of the manuscript, agreed to be accountable for all aspects of the work in ensuring that questions related to the accuracy or integrity of any part of the work were appropriately investigated and resolved.

## Conflict of Interest Statement

The authors declare that the research was conducted in the absence of any commercial or financial relationships that could be construed as a potential conflict of interest.
